# Social media use and abuse: Different profiles of users and their associations with addictive behaviours

**DOI:** 10.1016/j.abrep.2023.100479

**Published:** 2023-01-21

**Authors:** Deon Tullett-Prado, Vasileios Stavropoulos, Rapson Gomez, Jo Doley

**Affiliations:** a Victoria University, Australia; b University of Athens, Greece; c Federation University, Australia

**Keywords:** Social media addiction, Behavioral addiction, Substance addiction, Latent Profile Analysis, Analysis of Variance

## Abstract

**Introduction:**

Social media use has become increasingly prevalent worldwide. Simultaneously, concerns surrounding social media abuse/problematic use, which resembles behavioural and substance addictions, have proliferated. This has prompted the introduction of ‘Social Media Addiction’ [SMA], as a condition requiring clarifications regarding its definition, assessment and associations with other addictions. Thus, this study aimed to: (a) advance knowledge on the typology/structure of SMA symptoms experienced and: (b) explore the association of these typologies with addictive behaviours related to gaming, gambling, alcohol, smoking, drug abuse, sex (including porn), shopping, internet use, and exercise.

**Methods:**

A sample of 968 [Mage = 29.5, SDage = 9.36, nmales = 622 (64.3 %), nfemales = 315, (32.5 %)] adults was surveyed regarding their SMA experiences, using the Bergen Social Media Addiction Scale (BSMAS). Their experiences of Gaming, Internet, Gambling, Alcohol, Cigarette, Drug, Sex, Shopping and Exercise addictions were additionally assessed, and latent profile analysis (LPA) was implemented.

**Results:**

Three distinct profiles were revealed, based on the severity of one’s SMA symptoms: ‘low’, ‘moderate’ and ‘high’ risk. Subsequent ANOVA analyses suggested that participants classified as ‘high’ risk indicated significantly higher behaviours related to internet, gambling, gaming, sex and in particular shopping addictions.

**Conclusions:**

Results support SMA as a unitary construct, while they potentially challenge the distinction between technological and behavioural addictions. Findings also imply that the assessment of those presenting with SMA behaviours, as well as prevention and intervention targeting SMA at risk groups, should consider other comorbid addictions.

## 1 Introduction

Social media – a form of online communication in which users create profiles, generate and share content, while forming online social networks/communities (Obar & Wildman, 2015), is quickly growing to become almost all consuming in the media landscape. Currently the number of daily social media users exceeds 53 % (∼4.5 billion users) of the global population, approaching 80 % among more developed nations (Countrymeters, 2021, DataReportal, 2021). Due to technological advancements, the rise of ‘digital natives’ (i.e. children and adolescents raised with and familiarised with digital technology) and coronavirus pandemic triggered lockdowns, the frequency and duration of social media usage has been steadily increasing as people compensate for a lack of face to face interaction or grow with Social Media as a normal part of their lives (i.e. ∼ 2 h and 27 min average daily; DataReportal, 2021, Heffer et al., 2019, Zhong et al., 2020, Nguyen, 2021). Furthermore, social media is increasingly involved in various domains of life including education, economics and even politics, to the point where engagement with the economy and wider society almost necessitates its use, driving the continued proliferation of social media use (Calderaro, 2018, Nguyen, 2021, Mabić et al., 2020, Mourão and Kilgo, 2021). This societal shift towards increased social media use has had some positive benefits, serving to facilitate the creation and maintenance of social groups, increase access to opportunities for career advancement and created wide ranging and accessible education options for many users (Calderaro, 2018, Prinstein et al., 2020, Bouchillon, 2020, Nguyen, 2021). However, for a minority of users - roughly 5–10 % (Bányai et al., 2017, Luo et al., 2021, Brailovskaia et al., 2021) – social media use has become excessive, to the point where it dominates one’s life, similarly to an addictive behaviour - a state known as 'problematic social media use' (Sun & Zhang, 2020). For these users, social media is experienced as the single most important activity in one’s life, while compromising their other roles and obligations (e.g. family, romance, employment; Sun and Zhang, 2020, Griffiths and Kuss, 2017). This is a situation associated with low mood/depression, the compromise of one’s identity, social comparison leading to anxiety and self-esteem issues, work, academic/career difficulties, compromised sleep schedules and physical health, and even social impairment leading to isolation (Anderson et al., 2017, Sun and Zhang, 2020, Gorwa and Guilbeault, 2020).

### 1.1 Problematic social media engagement in the context of addictions

Problematic social media use is markedly similar to the experience of substance addiction, thus leading to problematic social media use being modelled by some as a behavioural addiction - social media addiction (SMA; Sun and Zhang, 2020). In brief, an addiction loosely refers to a state where an individual experiences a powerful craving to engage with a behaviour, and inability to control their related actions, such that it begins to negatively impact their life (Starcevic, 2016). Although initially the term referred to substance addictions induced by psychotropic drugs (e.g., amphetamines), it later expanded to include behavioural addictions (Chamberlain et al., 2016). These reflect a fixation and lack of control, similar to those experienced in the abuse of substances, related to one’s excessive/problematic behaviours (Starcevic, 2016).

Indeed, behavioural addictions, such as gaming, gambling and (arguably) social media addiction (SMA) share many common features with substance related addictions (Zarate et al., 2022). Their similarities extend beyond the core addiction manifestations of fixation, loss of control and negative life consequences (Grant et al., 2010, Bodor et al., 2016, Martinac et al., 2019, Zarate et al., 2022). For instance, it has been evidenced that common risk factors/mechanisms (e.g., low impulse control), behavioural patterns (e.g., chronic relapse; sudden “spontaneous” quitting), ages of onset (e.g., adolescence and young adulthood) and negative life consequences (e.g., financial and legal difficulties) are similar between the so-called behavioural addictions and formally diagnosed substance addictions (Grant et al., 2010). Moreover, such commonalities often accommodate the concurrent experience of addictive presentations, and/or even the substitution/flow from one addiction to the next (e.g., gambling and alcoholism; Bodor et al., 2016, Martinac et al., 2019, Grant et al., 2010).

With these features in mind, SMA has been depicted as characterized by the following six symptoms; A deep preoccupation with social media use (salience), use to either increase their positive feelings and/or buffer their negative feelings (mood modification), the requirement for progressively increasing time-engagement to get the same effect (i.e., tolerance), withdrawal symptoms such as irritability and frustration when access is reduced (withdrawal), the development of tensions with other people due to under-performance across several life domains (conflict) and reduced self-regulation resulting in an inability to reduce use (relapse; Andreassen et al., 2012, Brown, 1993, Griffiths and Kuss, 2017, Sun and Zhang, 2020).

This developing model of SMA has been gaining popularity as the most widely used conceptualisation of problematic social media use, and guiding the development of relevant measurement tools (Andreassen et al., 2012, Haand and Shuwang, 2020, Prinstein et al., 2020; Van den Eijnden et al., 2016)). However, SMA is not currently uniformly accepted as an understanding of problematic social media use. Some critics have labelled the SMA model a premature pathologisation of ordinary social media use behaviours with low construct validity and little evidence for its existence, often inviting alternative proposed classifications derived by cognitive-behavioural or contextual models (Sun & Zhang, 2020; Panova & Carbonell, 20187; Moretta, Buodo, Demetrovics & Potenza, 2022). Furthermore, the causes, risk factors and consequences of SMA, as well as the measures employed in its assessment have yet to be elucidated in depth, with research in the area being largely exploratory in nature (Prinstein et al., 2020, Sun and Zhang, 2020). In this context, what functional, regular and excessive social media use behaviours may involve has also been debated (Wegmann et al., 2022). Thus, there is a need for further research clarifying the nature of SMA, identifying risk factors and related negative outcomes, as well as potential methods of treatment (Prinstein et al., 2020, Sun and Zhang, 2020, Moretta et al., 2022).

Two avenues important for realizing these goals (and the focus of this study) involve: a) profiling SMA behaviours in the broader community, and b) decoding their associations with other addictions. Profiling these behaviours would involve identifying groups of people with particular patterns of use rather than simply examining trends in behaviour across the greater population. This would allow for clearer understandings of the ways in which different groups experience SMA and a more person-centred analysis (i.e., focused on finer understandings of personal experiences, Bányai et al., 2017). Moreover, when combined with analyses of association, it can allow for assertions not only about whether SMA associates with a variable, but about which components of the experience of SMA associate with a variable, allowing for more nuanced understandings. One such association with much potential for exploration, is that of SMA with other addictions (i.e., how does a certain SMA type differentially relate with other addictive behaviors, such as gambling and/or substance abuse?). Such knowledge would be useful, due to the shared common features and risk factors between addictions. It would allow for a greater understanding of the likelihood of comorbid addictions, or of flow from one addiction to the next (Bodor et al., 2016, Martinac et al., 2019, Grant et al., 2010). However, the various links between different addictions are not identical, with alcoholism (for example) associating less strongly with excessive/problematic internet use than with problematic/excessive (so called “addictive) sex behaviours (Grant et al., 2010). In that line, some studies have suggested the consideration of different addiction subgroups (e.g., substance, behavioural and technology addictions Marmet et al., 2019), and/or different profiles of individuals being prone to manifest some addictive behaviours more than others (Zilberman et al., 2018). Accordingly, one may assume that distinct profiles of those suffering from SMA behaviours may be more at risk for certain addictions over others, rather than with addictions in general (Zarate et al., 2022).

Understanding these varying connections could be vital for SMA treatment. Co-occurring addictions often reinforce each-other through their behavioural effects. Furthermore, by targeting only a single addiction type in a treatment, other addictions an individual is vulnerable to can come to the fore (Grant et al., 2010, Miller et al., 2019). Thus, a holistic view of addictive vulnerability may require consideration (Grant et al., 2010, Miller et al., 2019). This makes the identification of individual SMA profiles, as well as any potential co-occurring addictions, pivotal for more efficient assessment, prevention and intervention of SMA behaviours.

To the best of the authors’ knowledge, four studies to date have attempted to explore SMA profiles. Three of those have been conducted predominantly with European adolescent samples, and varied in terms of the type and number of profiles detected (Bányai et al., 2017, Brailovskaia et al., 2021, Luo et al., 2021, Cheng et al., 2022). The fourth was conducted with English speaking adults from the United Kingdom and the United States (Cheng et al., 2022). Of extant studies, Bányai et al. (2017) identified three profiles varying quantitively (i.e., in terms of their SMA symptoms’ severity) across a low, moderate and high range. In contrast, Brailovskaia et al., 2021, Luo et al., 2021 identified four and five profiles that varied both quantitatively and qualitatively in terms of the type of SMA symptoms reported. Brailovskaia et al., (2021) proposed the ‘low symptom’, ‘low withdrawal’ (i.e., lower overall SMA symptoms with distinctively lower withdrawal), ‘high withdrawal’ (i.e., higher overall SMA symptoms with distinctively higher withdrawal) and ‘high symptom’ profiles. Luo et al. (2021) supported the ‘casual’, ‘regular’, ‘low risk high engagement’, ‘at risk high engagement’ and ‘addicted’ user profiles, which demonstrated progressively higher SMA symptoms severity alongside significant differences regarding mood modification, relapse, withdrawal and conflict symptoms, that distinguished the low and high risk ‘high engagement’ profiles. Finally, considering the occurrence of different SMA profiles in adults, Cheng and colleagues, (2022), supported the occurrence of ‘no-risk’, ‘at risk’ and ‘high risk’ social media users applying in both US and UK populations, with the UK sample showing a lower proportion of the ‘no-risk’ profile (i.e. UK = 55 % vs US = 62.2) and a higher percentage of the high risk profile (i.e. UK = 11.9 % vs US = 9.1 %). Thus, considering the number of identified profiles best describing the population of social media users, Cheng and colleagues’ findings (2022) were similar to Bányai and colleagues’ (2017) suggestions for SMA behaviour profiles of adolescents. At this point it should be noted, that none of the four studies exploring SMA behaviours profiles to date has taken into consideration different profile parameterizations, meaning that potential differences in the heterogeneity/ variability of those classified within the same profile were not considered (e.g. some profiles maybe more loose/ inclusive than others; Bányai et al., 2017, Brailovskaia et al., 2021, Luo et al., 2021, Cheng et al., 2022).

The lack of convergence regarding the optimum number and the description of SMA profiles occurring, as well as age, cultural and parameterization limitations of the four available SMA profiling studies, invites further investigation. This is especially evident in light of preliminary evidence confirming one’s SMA profile may link more to certain addictions over others (Zarate et al., 2022). Indeed, those suffering from SMA behaviours have been shown to display heightened degrees of alcohol and drug use, a vulnerability to internet addiction in general, while presenting lower proneness towards exercise addiction and tobacco use (Grant et al., 2010, Anderson et al., 2017, Duradoni et al., 2020, Spilkova et al., 2017). In terms of gambling addiction, social media addicts display similar results on tests of value-based decision making as gambling addicts (Meshi et al., 2019). Finally, regarding shopping addiction, the proliferation of advertisements for products online, and the ease of access via social media to online stores could be assumed to have an intensifying SMA effect (Rose & Dhandayudham, 2014). Aside from these promising, yet relatively limited findings, the assessed connections between SMA and other addictions tend to be either addressed in isolation (e.g., SMA with gambling only and not multiple other addiction forms; Gainsbury et al., 2016a, Gainsbury et al., 2016b) and in a variable (and not person) focused manner (e.g., higher levels of SMA relate with higher levels of drug addiction; Spilkova et al., 2017), which overlooks an individual’s profile. These profiles are vitally needed, as knowing the type of individual who may experience a series of disparate addictions is paramount for identifying at risk social media users and populations in need of more focused prevention/intervention programs (Grant et al., 2010). Hence, using person focused methods such as latent profile(s) analysis (LPA) that address the ways in which distinct variations/profiles in SMA behaviours may occur, and how these relate with other addictions is imperative (Lanza & Cooper, 2016).

### 1.2 Present study

To address this research priority, while considering SMA behaviours as being normally distributed (i.e., a minimum–maximum continuum) across the different profiles of users in the general population, the present Australian study uses a large community sample, solid psychometric measures and a sequence of differing in parameterizations LCA models aiming to: (a) advance past knowledge on the typology/structure of SMA symptom one experiences and: (b) innovatively explore the association of these typologies with a comprehensive list of addictive behaviours related to gaming, gambling, alcohol, smoking, drug abuse, sex (including porn), shopping, internet use, and exercise.

Based on Cheng and colleagues (2022) and Bányai and colleagues (2017), it was envisaged that three profiles arrayed in terms of ascending SMA symptoms’ severity would be likely identified. Furthermore, guided by past literature supporting closer associations between technological and behavioural addictions than with substance related addictions, it was hypothesized that those classified at higher SMA risk profiles would report higher symptoms of other technological and behavioural addictions, such as those related to excessive gaming and gambling, than with drug addiction (Chamberlain and Grant, 2019, Zarate et al., 2022).

## 2 Method

### 2.1 Participants

The current study was conducted in Australia. Responses initially retrieved included 1097 participants. Of those, 129 were not considered for the current analyses. In particular, 84 respondents were classified as preview-only registrations and did not address any items, 5 presented with systematic response inconsistencies, and thus were considered invalid, 11 were excluded as potential bots, 11 had not provided their informed consent (i.e., did not tick the digital consent box, although they later addressed the survey), and 18 were taken out for not fulfilling age conditions (i.e., being adults), in line with the ethics approval received. Therefore, responses from 968 English-speaking adults from the general community were examined. An online sample of adult, English speaking participants aged 18 to 64 who were familiar with gaming [*N* = 968, *M*_age_ = 29.5, *SD*_age_ = 9.36, *n*_males_ = 622 (64.3 %), *n*_females_ = 315, (32.5 %), *n_trans/non-binary_ = 26* (2.7 %), *n_queer_ =* 1 (0.1 %), *n_other_ =* 1 (0.1 %), *n_missing_ =* 3 (0.3 %)] was analysed. According to Hill (1998) random sampling error is required to lie below 4 %, that is satisfied by the current sample’s 3 % (SPH analytics, 2021). See Table 1 for participants’ sociodemographic information.

**Table 1:** Socio-demographic and online use characteristics of participants.

**Sociodemographic variables**		**Males** ***n***	**%**	**Females** ***n***	**%**	**Nonbinary/Other** ***n***	**31** **%**
Ethnicity	White/Caucasian	380	61.1	193	61.2	22	71
	Black/African American	31	5	23	7.3	1	3.2
	Asian	124	19.9	59	18.7	1	3.2
	Hispanic/Latino	35	5.6	9	2.9	2	6.4
	Other (Aboriginal, Indian, Pacific Islander, Middle eastern, Mixed, other)	52	8.3	31	9.8	5	16.1
Sexual Orientation	Heterosexual/Straight	529	85.5	211	67	3	9.7
	Homosexual/Gay	33	5.3	13	4.1	4	12.9
	Bisexual	48	7.7	65	20.6	11	35.5
	Other	12	1.9	26	8.3	12	38.7
Employment status	Full Time	238	38.3	86	27.3	7	22.6
	Part Time/Casual	73	12.7	60	19	1	3.2
	Self Employed	48	7.7	17	5.4	2	6.4
	Unemployed	125	20.1	60	21.2	7	22.6
	Student/Other	138	22.2	92	23.8	14	45.2
Level of Education	Elementary/Middle school	10	1.6	2	0.6	0	0
	High School or equivalent	166	26.7	74	23.5	11	35.5
	Vocational/Technical School/Tafe	55	8.8	26	8.3	4	12.9
	Some Tertiary Education	113	18.2	69	21.9	3	9.7
	Bachelor’s Degree (3 years)	137	22	76	24.1	5	16.1
	Honours Degree or Equivalent (4 years)	69	11.1	35	11.1	5	16.1
	Masters Degree (MS)	47	7.6	20	6.3	1	3.2
	Doctoral Degree (PhD)	4	0.6	4	1.3	1	3.2
	Other/Prefer not to say	21	3.3	9	2.8	1	3.2
Marital/Relationship status	Single	405	65.1	164	52.1	23	74.2
	Partnered	68	10.9	62	19.7	7	22.6
	Married	120	19.3	68	21.6	0	0
	Separated	15	2.4	14	4.4	0	0
	Other/Prefer not to say	14	2.2	7	2.2	1	3.2
		

Note: Percentages represent the percentage of that sex which is represented by any one grouping, rather than percentages of the overall population.

### 2.2 Measures

Psychometric instruments targeting sociodemographics, SMA and a semi-comprehensive range of behavioral, digital and substance addictions were employed. These instruments involved the Bergen Social Media Addiction Scale (BSMAS; Andreassen et al., 2012), the Internet Gaming Disorder 9 items Short Form (IGDS-SF9; Pontes & Griffiths, 2015), The Internet Disorder Scale (IDS9-SF; (Pontes & Griffiths, 2016), the Online Gambling Disorder Questionnaire (IGD-Q; González-Cabrera et al., 2020), the 10-Item Alcohol Use Disorders Identification Test (AUDIT; Saunders et al., 1993, the Five Item Cigarette Dependance Scale (CDS-5; Etter et al., 2003), the 10- item Drug Abuse Screening Test (DAST-10; Skinner, 1982), the Bergen-Yale Sex Addiction Scale (BYSAS; Andreassen et al., 2018), the Bergen Shopping Addiction Scale (BSAS; Andreassen et al., 2015) and the 6-item Revised Exercise Addiction Inventory (EAI-R; Szabo et al., 2019). Precise details of these measures, including values related to assumptions can be found in Table 2.

**Table 2:** Measure descriptions and internal consistency.

**Name & Abbreviation**	Instrument’s Description	Reliability in the current data (α and ω)	Normality Distribution in the current data
The Bergen Social Media Addiction Scale (BSMAS)	The BSMAS measures the severity of one’s experience of Social Media Addiction (SMA) symptoms (i. e. salience, mood, modification, tolerance, withdrawal conflict and relapse; Andreassen et al., 2012). These are measured using six questions relating to the rate at which certain behaviours/states are experienced. Items are scored from 1 (very rarely) to 5 (very often) with higher scores indicating a greater experience of SMA Symptoms (Andreassen et al., 2012).	α = 0.88. ω = 0.89.	Skewness = 0.89 Kurtosis = 0.26
The Internet Gaming Disorder 9 items Short Form (IGDS-SF9)	The IGDS-SF9 measures the severity of one’s disordered gaming behaviour on each of the 9 DSM-5 proposed criteria (e.g. Have you deceived any of your family members, therapists or others because the amount of your gaming activity?”(Pontes & Griffiths, 2015). Items are addressed following a 5-point Likert scale ranging from 1 (Never) to 5 (very often). Responses are accrued informing a total score ranging from 9 to 45 with higher scores indicating higher disordered gaming manifestations.	α = 0.88. ω = 0.89.	Skewness = 0.94 Kurtosis = 0.69
The Internet Disorder Scale – Short form (IDS9-SF)	Measures the severity of one’s experience of excessive internet use as measured by nine symptom criteria/items adapted from the DSM-5 disordered gaming criteria (e. g. “Have you deceived any of your family members, therapists or other people because the amount of time you spend online?”; Pontes & Griffiths, 2016). The nine items are scored via a 5-point Likert scale ranging from 1 (Never) to 5 (very often) with higher scores indicating more excessive internet use.	α = 0.90. ω = 0.90.	Skewness = 0.74 Kurtosis = 0.11
The Online Gambling Disorder Questionnaire (OGD-Q)	Measures the degree to which one’s online gambling behaviours have become problematic (González-Cabrera et al., 2020). It consists of 11 items asking about the rate certain states or behaviours related to problematic online gambling are experienced in the last 12 months (e.g. Have you felt that you prioritized gambling over other areas of your life that had been more important before?). Responses are addressed on a 5-point Likert scale ranging from 0 (never) to 4 (Every day) with a higher aggregate score indicating greater risk of Gambling Addiction.	α = 0.95. ω = 0.95.	Skewness = 3.45 Kurtosis = 13.90
The 10-Item Alcohol Use Disorders Identification Test (AUDIT)	Screens potential problem drinkers for clinicians (Saunders et al., 1993). Comprised of 10 items scored on a 5-point Likert scale, the AUDIT asks participants questions related to the quantity and frequency of alcohol imbibed, as well as certain problematic alcohol related states/behaviours and the relationship one has with alcohol (e.g. Have you or someone else been injured as a result of you drinking?). Items are scored on a 5 point Likert scale, however due to the varying nature of these questions, the labels used on these responses vary. Higher scores indicate a greater risk, with a score of 8 generally accepted as a dependency indicative point.	α = 0.89. ω = 0.91.	Skewness = 2.13 Kurtosis = 4.84
The Five Item Cigarette Dependence Scale (CDS-5)	Measures the five DSM-IV and ICD-11 dependence criteria in smokers (Etter et al., 2003). It features 5 items enquiring into specific aspects of cigarette dependency such as cravings or frequency of use, answered via a 5-point Likert scale (e. g. Usually, how soon after waking up do you smoke your first cigarette?). Possible response labels vary to follow the different questions’ phrasing/format (e.g. frequencies, subjective judgements, ease of quitting;Etter et al., 2003).	α = 0.68. ω = 0.87.	Skewness = 1.52 Kurtosis = 2.52
The 10-item Drug Abuse Screening Test (DAST-10)	Screens out potential problematic drug users (Skinner, 1982). It features 10 items asking yes/no questions regarding drug use, frequency and dependency symptoms (e.g. Do you abuse more than one drug at a time?). Items are scored “0″ or “1” for answers of “no” or “yes” respectively, with higher aggregate scores indicating a higher likelihood of Drug Abuse and a proposed cut-off score between 4 and 6.	α = 0.79. ω = 0.88.	Skewness = 2.49 Kurtosis = 6.00
The Bergen-Yale Sex Addiction Scale (BYSAS)	Measures sex addiction on the basis of the behavioural addiction definition (Andreassen et al., 2018). It features six items enquiring about the frequency of certain actions/states (e.g. salience, mood modification), rated on a 5-point Likert scale ranging from 0 (Very rarely) to 4 (Very often).	α = 0.84. ω = 0.84.	Skewness = 0.673 Kurtosis = 0.130
The Bergen Shopping Addiction Scale (BSAS)	Measures shopping addiction on the basis of seven behavioural criteria (Andreassen et al., 2015). These 7 items enquire into the testee’s agreement with statements about the frequency of certain shopping related actions/states (e.g. I feel bad if I for some reason am prevented from shopping/buying things”) rated on a 5-point Likert scale ranging from 1 (Completely disagree) to 5 (Completely agree). Greater aggregate scores indicate an increased risk of shopping addiction.	α = 0.88. ω = 0.89.	Skewness = 0.889 Kurtosis = 0.260
The 6-item Revised Exercise Addiction Inventory (EAI-R)	Assesses exercise addiction, also on the basis of the six behavioural addiction criteria through an equivalent number of items (Szabo et al., 2019). It comprises six statements about the relationship one has with exercise (e.g. Exercise is the most important thing in my life) rated on a 5-point likert scale ranging from 1 (Strongly Disagree) to 5 (Strongly agree) and higher aggregate scores indicating a higher risk.	α = 0.84. ω = 0.84.	Skewness = 0.485 Kurtosis = -0.451

*Note*Table 2*:* Streiner’s (2003) guidelines are used when measuring internal reliability, with Cronbachs Alpha scores in the range of 0.60–0.69 labelled ‘acceptable’, ranges between 0.70 and 0.89 labelled ‘good’ and ranges between 0.90 and 1.00 labelled ‘excellent’. Acceptable values of skewness fall between − 3 and + 3, and kurtosis is appropriate from a range of − 10 to + 10 (Brown, 2006). OGD-G kurtosis (13.90) and skewness (3.45) exceeded the recommended limits (Brown, 2006). However, LPA does not assume data distribution linearity, normality and or homogeneity (Rosenberg et al., 2019). Considering aim B, related to detecting significant reported differences on measures for gaming, sex, shopping, exercise, gambling, alcohol, drug, cigarette and internet addiction symptoms respectively, anova results were derived after bootstrapping the sample 1000 times to ensure that normality assumptions were met. Case bootstrapping calculates the means of 1000 resamples of the available data and computes the results analysing these means, which are normally distributed (Tong, Saminathan, & Chang, 2016).

### 2.3 Procedure

Approval was received from the Victoria University Human Research Ethics Committee (HRE20-169). Data was collected in August 2019 to August 2020 via an online survey link distributed via social media (i.e., Facebook; Instagram; Twitter), digital forums (i.e. reddit) and the Victoria University learning management system. Familiarity with gaming was preferred, so that associations with one’s online gaming patterns were studied. The link first took potential participants to the Plain Language Information Statement (PLIS) which informed on the study requirements and participants’ anonymity and free of penalty withdrawal rights. Digital provision of informed consent (i.e., ticking a box) was required by the participants before proceeding to the survey.

### 2.4 Statistical analyses

Statistical analyses were conducted via: a) R-studio for the latent profile(s) analyses (LPA) and; b) Jamovi for descriptive statistics and profiles’ comparisons. Regarding aim A, LPA identified naturally homogenous subgroups within a population (Rosenberg et al., 2019). Through the TIDYLPA CRAN R package, a number of models varying in terms of their structure/parameterization and the number of ‘profiles’ were tested using the six BSMAS criteria/items as indicators (Rosenberg et al., 2019; see Table 3).

**Table 3:** LCA model parameterization characteristics.

Model Number	Means	Variances	Covariances		Interpretation
Class-Invariant Parameterization (CIP)	Varying	Equal	Zero		Different classes/profiles have different means on BSMAS symptoms. Despite this, the differences of the minimum and maximum rates for the six BSMAS symptoms do not significantly differ across the classes/profiles. Finally, there is no covariance in relation to the six BSMAS symptoms across the profiles.
Class-Varying Diagonal Parameterization (CVDP)	Varying	Varying	Zero		Different classes/profiles have different means on BSMAS symptoms but similar differences between their minimum and maximum scores. Additionally, there is an existing similar pattern of covariance considering the six BSMAS symptoms across the classes.
Class-Invariant Unrestricted Parameterization (CIUP)	Varying	Equal	Equal		Different classes in the model have different means on the six BSMAS symptoms. The range between the minimum and maximum scores of the six BSMAS symptoms is dissimilar across the profiles. Last, there is differing covariance based on the six BSMAS symptoms across the classes.
Class-Varying Unrestricted Parameterization (CVUP)	Varying	Varying	Varying		Different classes in the model have different means on the six BSMAS symptom. The range between the minimum and maximum scores of the six BSMAS symptoms is dissimilar across the profiles. Last, there is differing covariance based on the six BSMAS symptoms across the classes.

Subsequently, the constructed models were compared regarding selected fit indices (i.e., Akaike Information Criterion (AIC) and the Bayesian Information Criterion (BIC), bootstrapped Lo-Mendel Rubin test (B-LMR or LRT), entropy and the N_Min; Rosenberg et al., 2019)^1^. This involved 1: Dismissing any models with *N*-Min’s equalling 0, as each profile requires at least one participant, 2: Dismissing models with entropy scores below 0.64 (Tein et al., 2013), 3: Dismissing models with nonsignificant BLMR value, and 4: assessing the remaining models on their AIC/BIC looking for an elbow point in the decline or the lowest values.

Regarding aim B of the study, ANOVA with bootstrapping (1000x) was employed to detect significant profile differences regarding one’s gaming, sex, shopping, exercise, gambling, alcohol, drug, cigarette and internet addiction symptoms respectively.

## 3 Results

All analyses’ assumptions were met with one exception^2^. The measure of Online Gambling disorder experience violated guidelines for the acceptable departure from normality and homogeneity (Kim, 2013). Given this violation, results regarding gambling addiction should be considered with some caution.

### 3.1 Aim A: LPA of BSMAS symptoms

The converged models’ fit, varying by number of profiles and parametrization is displayed in Table 4, with the CIP parameterisation presenting as the optimum (i.e. lower AIC and BIC, and 1–8 profiles converging; all CVDP, CIUP, CVUP models did not converge except the CVUP one profile). Subsequently, the CIP models were further examined via the TIDYLPA Mclust function (see Table 5). AIC and BIC decreased as the number of profiles increased. This flattened past 3 profiles (i.e., elbow point; Rosenberg et al., 2019). Furthermore, past 3 profiles, *N*-min reached zero, indicating profiles with zero participants in them – thus reducing interpretability. Lastly, the BLRT test reached non significance once the model had 4 profiles, again indicating the 3-profile model as best fitting. Therefore, alternative CIP -models were rejected in favour of the 3-profile one. This displayed a level of classification accuracy well above the suggested cut off point of 0.76 (entropy = 0.90; Larose et al., 2016), suggesting over 90 % correct classification (Larose et al., 2016). Regarding the profiles’ proportions, counts revealed 33.6 % as profile 1, 52.4 % as profile 2, 14 % as profile 3.

**Table 4:** Initial model testing.

Model	Classes	AIC	BIC
CIP	1	18137.5	18196.0
	2	15787.6	15880.2
	3	15040.5	15167.3
	4	15054.6	15215.4
	5	15068.7	15263.7
	6	14548.8	14778.0
	7	14562.8	14826.1
	8	14350.1	14647.5
CVUP	1	15218.2	15349.8

**Table 5:** Fit indices of cip models with 1–8 classes.

Model	Classes	AIC	BIC	Entropy	n_min	BLRT_p
CIP	1	18137.6	18196.1	1	1	
CIP	2	15780.5	15873.1	0.89	0.35	0.01
CIP	3	15025.3	15152.1	0.90	0.14	0.01
CIP	4	15039.4	15200.2	79	0	1
CIP	5	15053.7	15248.7	0.7	0	1
CIP	6	14777.7	15006.8	0.77	0	0.01
CIP	7	14557.6	14820.9	0.8	0	0.01
CIP	8	14449.9	14747.2	0.81	0	0.01

**Fig. 1:**
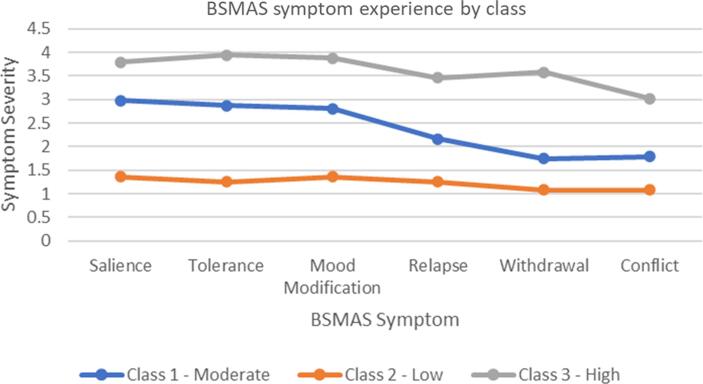
Raw symptom experience of the three classes.

**Fig. 2:**
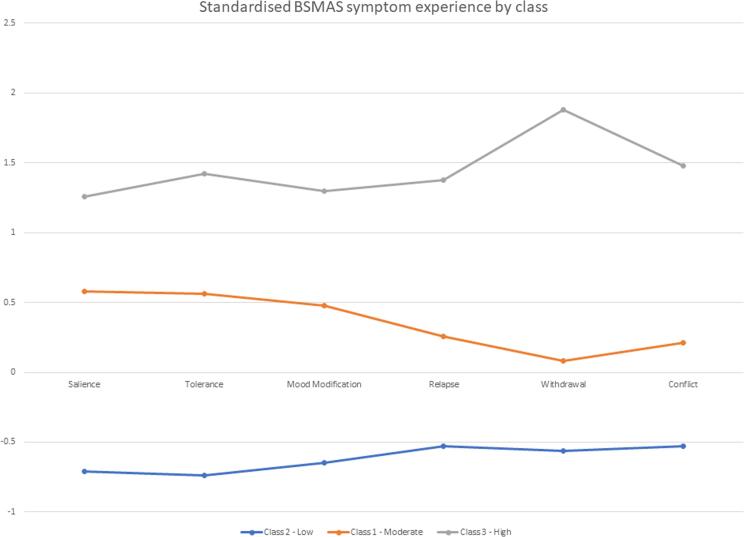
Standardized symptom experience of the three classes.

Table 6 and Fig. 1 present the profiles’ raw mean scores across the 6 BSMAS items whilst Table 7 and Fig. 2 present the standardised mean scores.

**Table 6:** Raw Mean Scores and Standard Error of the 6 BSMAS Criteria Across the Three Classes/Profiles.

Symptom Class	Salience	Tolerance	Mood Modification	Relapse	Withdrawal	Conflict
1	2.98	2.87	2.81	2.16	1.74	1.79
2	1.36	1.25	1.36	1.25	1.08	1.08
3	3.8	3.95	3.88	3.46	3.58	3.02
SE (Equal across classes)	0.07	0.07	0.08	0.08	0.09	0.08

**Table 7:** Standardised mean scores of the 6 bsmas criteria Across the Three Classes/Profiles.

Symptom Class	Salience	Tolerance	Mood Modification	Relapse	Withdrawal	Conflict
1	0.58	0.56	0.48	0.26	0.08	0.21
2	−0.71	−0.74	−0.65	−0.53	−0.56	−0.53
3	1.26	1.42	1.30	1.38	1.88	1.48

Note: For standard errors, see Table 6.

Profile 1 scores varied from 1.74 to 2.98 raw and between 0.08 and 0.58 standard deviations above the sample mean symptom experience. In terms of plateaus and steeps, profile 1 displayed a raw score plateaus across symptoms 1–3 (salience, tolerance, mood modification), a decline in symptom 4 (relapse), and another plateau across symptoms 5–6 (withdrawal and conflict). It further displayed a standardized score plateau around the level of 0.5 standard deviations across symptoms 1–3 and a decline across symptoms 4–6. Profile 2 varied consistently between raw mean scores of 1 and 1.36 across the 6 SMA symptoms, and between −0.74 and −0.53 standard deviations from the sample mean with general plateaus in standardized score across symptoms 1–3 and 4–6. Finally, profile 3 mean scores varied between 3.02 and 3.95 raw and 1.26 to 1.88 standardized. Plateaus were witnessed in the raw scores across symptoms 1–3 (salience, tolerance, mood modification), a decline at symptom 4 (relapse), a relative peak at symptom 5 (withdrawal), and a further decline across symptom 6 (conflict). However, the standardized scores for profile 3 were relatively constant across the first four symptoms, before sharply reaching a peak at symptom 5 and then declining once more. Accordingly, the three profiles were identified as severity profiles ‘Low’ (profile 2), ‘Moderate’ (profile 1) and ‘High’ (profile 3) risk. Table 8, Table 9 provide the profile means and standard deviations, as well as their pairwise comparisons across the series of other addictive behaviors assessed.

**Table 8:** Post Hoc Descriptives across a semi-comprehensive list of addictions.

Comparison/Class	Mean	Standard Deviation	N
**Gaming Addiction**			
Low	16.216	6.353	501
Moderate	19.186	6.655	322
High	22.216	8.124	134

**Alcoholism**			
Low	3.877	5.175	503
Moderate	4.491	6.034	324
High	6.610	8.018	136

**Smoking**			
Low	9.264	4.134	507
Moderate	9.028	3.725	325
High	9.551	3.955	136

**Drug Use**			
Low	1.561	1.513	506
Moderate	1.754	1.787	325
High	2.044	1.881	136

**Sex Addiction**			
Low	5.568	4.640	505
Moderate	7.115	4.898	323
High	9.687	5.769	134

**Shopping addiction**			
Low	11.565	4.829	503
Moderate	14.804	5.173	321
High	17.993	7.222	134

**Exercise Addiction**			
Low	13.812	6.467	500
Moderate	14.646	6.009	322
High	15.793	7.470	135

**Gambling Addictions**			
Low	12.261	3.178	502
Moderate	14.270	6.190	315
High	16.948	9.836	135

**Internet Addiction**			
Low	17.022	7.216	501
Moderate	21.165	6.554	321
High	27.971	7.340	136

**Table 9:** Post Hoc Comparisons of the SMA profiles revealed across the addictive behaviors measured.

Comparison/Class	Mean Difference		SE		t		p_tukey_
**Gaming Addiction**							
Low vs moderate	−2.971		0.481		−6.183		< 0.001
Low vs High	−6.650		0.654		−10.164		< 0.001
Moderate vs High	−3.679		0.692		−5.320		< 0.001

**Alcoholism**							
Low vs moderate	−0.614		0.423		−1.451		0.315
Low vs High	−2.734		0.574		−4.761		< 0.001
Moderate vs High	−2.120		0.607		−3.492		0.001

**Smoking**							
Low vs moderate	0.237		0.283		0.837		0.680
Low vs High	−0.287		0.384		−0.748		0.735
Moderate vs High	−0.524		0.406		−1.290		0.401

**Drug Use**							
Low vs moderate	−0.193		0.118		−1.628		0.234
Low vs High	−0.483		0.161		−3.005		0.008
Moderate vs High	−0.290		0.170		−1.708		0.203

**Sex Addiction**							
Low vs moderate	−1.546		0.349		−4.431		< 0.001
Low vs High	−4.118		0.476		−8.653		< 0.001
Moderate vs High	−2.572		0.503		−5.111		< 0.001

**Shopping addiction**							
Low vs moderate	−3.239		0.381		−8.495		< 0.001
Low vs High	−6.428		0.519		−12.387		< 0.001
Moderate vs High	−3.189		0.549		−5.809		< 0.001

**Exercise Addiction**							
Low vs moderate	−0.834		0.462		−1.804		0.169
Low vs High	−1.981		0.628		−3.156		0.005
Moderate vs High	−1.147		0.663		−1.728		0.195

**Gambling Addictions**							
Low vs moderate	−2.009		0.405		−4.966		< 0.001
Low vs High	−4.687		0.546		−8.591		< 0.001
Moderate vs High	−2.678		0.579		−4.626		< 0.001

**Internet Addiction**							
Low vs moderate	−4.143		0.502		−8.256		< 0.001
Low vs High	−10.949		0.679		−16.131		< 0.001
Moderate vs High	−6.805		0.718		−9.476		< 0.001

### 3.2 Aim 2: BSMAS profiles and addiction risk/personal factors.

Table 8, Table 9 display the Jamovi outputs for the BSMAS profiles and their means and standard deviations, as well as their pairwise comparisons across the series of other addictive behaviors assessed using ANOVA. Cohen’s (1988) benchmarks were used for eta squared values, with > 0.01 indicating small, >0.059 medium and > 0.138 large effects. ANOVA results were derived after bootstrapping the sample 1000 times to ensure that normality assumptions were met. Case bootstrapping calculates the means of 1000 resamples of the available data and computes the results analysing these means, which are normally distributed (Tong et al., 2016). SMA profiles significantly differed across the range of behavioral addiction forms examined with more severe SMA profiles presenting consistently higher scores with a medium effect size regarding gaming (*F* = 57.5, *p* <.001, η^2^ = 0.108), sex (*F* = 39.53, *p* <.001, η^2^ = 0.076) and gambling (*F* = 40.332, *p* <.001, η^2^ = 0.078), and large effect sizes regarding shopping (*F* = 90.06, *p* <.001, η^2^ = 0.159) and general internet addiction symptoms (*F* = 137.17, *p* <.001, η2 = 0.223). Only relationships of ‘medium’ size or greater were considered further in this analysis, though small effects were found with alcoholism (*F* = 11.34, *p* <.001, η^2^ = 0.023), substance abuse (*F* = 4.83, *p* =.008, η^2^ = 0.01) and exercise addiction (*F* = 5.415, *p* =.005, η2 = 0.011). Pairwise comparisons consistently confirmed that the ‘low’ SMA profile scored significantly lower than the ‘moderate’ and the ‘high’ SMA profile’, and the ‘moderate’ SMA profile being significantly lower than the ‘high’ SMA profile across all addiction forms assessed (see Table 8, Table 9).

## 4 Discussion

The present study examined the occurrence of distinct SMA profiles and their associations with a range of other addictive behaviors. It did so via uniquely combining a large community sample, measures of established psychometric properties addressing both SMA and an extensive range of other proposed substance and behavioral addictions, to calculate the best fitting model in terms of parameterization and profile number. A model of the CIP parameterization with three profiles was supported by the data. The three identified SMA profiles ranged in terms of severity and were labeled as ‘low’ (52.4 %), ‘moderate’ (33.6 %) and ‘high’ (14 %) SMA risk. Membership of the ‘high’ SMA risk profile was shown to link with significantly higher reported experiences of Internet and shopping addictive behaviours, and moderately with higher levels of addictive symptoms related to gaming, sex and gambling.

### 4.1 Number and variations of SMA profiles

Three SMA profiles, entailing ‘low’ (52.4 %), ‘moderate’ (33.6 %) and ‘high’(14 %) SMA risk were supported, with symptom 5 – withdrawal – displaying the highest inter-profile disparities. These results help clarify the number of SMA profiles in the population, as past findings were inconsistent supporting either 3, or 4 or 5 SMA profiles (Bányai et al., 2017, Brailovskaia et al., 2021, Luo et al., 2021), as well as the nature of the differences between these profiles (i.e. quantitative: “how much/high one experiences SMA symptoms” or qualitative: “the type of SMA symptoms one experiences”). Our findings are consistent with the findings of Bányai and colleagues (2017) and Cheng and colleagues (2022) indicating a unidimensional experience of SMA (i.e., that the intensity/severity an individual reports best defines their profile membership, rather than the type of SMA symptoms) with three profiles ranging in severity from ‘low’ to ‘moderate’ to ‘high’ and those belonging at the higher risk profiles being the minority. Conversely, these results stand in opposition with two past studies identifying profiles that varied qualitatively (i.e., specific SMA symptoms experienced more by certain profiles) and suggesting the occurrence of 4 and 5 profiles respectively (Brailovskaia et al., 2021, Luo et al., 2021). Such differences might be explained by variations in the targeted populations of these studies. Characteristics such as gender, nationality and age all have significant effects on how and why social media is employed (Andreassen et al., 2016; Hsu et al., 2015; Park et al., 2015). Given that the two studies in question utilized European, adolescent samples, the difference in the culture and age of our samples may have produced our varying results, (Brailovskaia et al., 2021, Luo et al., 2021). Comparability issues may also explain these results, given the profiling analyses implemented in the studies of Brailovskaia and colleagues, (2021), as well as Luo and colleagues (2021) did not extensively consider different profiles parameterizations, as the present study and Cheng et al. (2022) did. Furthermore, the results of this study closely replicated those of the Cheng et al., (2022) study, with both studies identifying a near identical pattern of symptom experience across three advancing levels of severity. This replication of results may indicate their accuracy, strengthening the validity of SMA experience models involving 3 differentiated profiles of staggered severity. Both our findings and Cheng et al.’s findings indicate profiles characterized by higher levels of cognitive symptoms (salience, withdrawal and mood modification) for each class when compared to their experience of behavioral symptoms (Relapse, withdrawal, conflict; Cheng et al., 2022). Further research may focus on any potentially mediating/moderating factors that may be interfering, and potentially further replicate such results, proving their reliability. Furthermore, given that past studies (with different results) utilized European, adolescent samples, cultural and age comparability limitations need to be considered and accounted for in future research (Bányai et al., 2017, Brailovskaia et al., 2021; Cheng et al., 2022).

Regarding withdrawal being the symptom of highest discrepancy between profiles, findings suggest that it may be more SMA predictive, and thus merit specific assessment or diagnostic attention, aligning with past literature (Bányai et al., 2017, Luo et al., 2021, Brailovskaia et al., 2021, Smith and Short, 2022). Indeed, the experience of irritability and frustration when abstaining from usage has been shown to possess higher differentiation power regarding diagnosing and measuring other technological addictions such as gaming, indicating the possibility of a broader centrality to withdrawal across the constellation of digital addictions (Gomez et al., 2019; Schivinski et al., 2018).

Finally, the higher SMA risk profile percentage in the current study compared with previous research [e.g., 14 % in contrast to the 4.5 % (Bányai et al., 2017), 4.2 % (Luo et al., 2021) and 7.2 % (Brailovskaia et al., 2021)] also invites significant plausible interpretations. The data collection for the present Australian study occurred between August 2019 to August 2020, while Bányai and their colleagues (2017) collected their data in Hungary in March 2015, and Brailovskaia and their colleagues (2021) in Lithuania and Germany between October 2019 and December 2019. The first cases of the COVID-19 pandemic outside China were reported in January 2020, and the pandemic isolation measures prompted more intense social media usage, to compensate for their lack of in person interactions started unfolding later in 2020 (Ryan, 2021, Saud et al., 2020). Thus, it is likely that the higher SMA symptom scores reported in the present study are inflated by the social isolation conditions imposed during the time the data was collected. Furthermore, the present study involves an adult English-speaking population rather than European adolescents, as the studies of Bányai and their colleagues (2017) and Brailovskaia and their colleagues (2021). Thus, age and/or cultural differences may explain the higher proportion of the high SMA risk profile found. For instance, it is possible that there may be greater SMA vulnerability among older demographics and/or across countries. The explanation of differences across counties is reinforced by the findings of Cheng and colleagues (2022) who assessed and compared UK and US adult populations, the first is less likely, as younger age has been shown to relate to higher SMA behaviors (Lyvers et al., 2019). Overall, the present results closely align with that of Cheng and colleagues (2022), who also collected their data during a similar period (between May 18, 2020 and May 24, 2020) from English speaking countries (as the present study did). They, in line with our findings, also supported the occurrence of three SMA behavior profiles, with the low risk profile exceeding 50 % of the general population and those at higher risk ranging above 9 %.

### 4.2 Concurrent addiction risk

Considering the second study aim, ascending risk profile membership was strongly related to increased experiences of internet and shopping addiction, while it moderately connected with gaming, gambling and sex addictions. Finally, it weakly associated with alcohol, exercise and drug addictions. These findings constitute the first semi-comprehensive cross-addiction risk ranking of SMA high-risk profiled individuals, allowing the following implications.

Firstly, no distinction was found between the so called “technological” and other behavioral addictions, potentially contradicting prior theory on the topic (Gomez et al., 2022). Typically, the abuse of internet gaming/pornography/social media, has been classified as behavioral addiction (Enrique, 2010, Savci and Aysan, 2017). However, their shared active substance – the internet – has prompted some scholars to suggest that these should be classified as a distinct subtype of behavioral addictions named “technological/ Internet Use addictions/disorders” (Savci & Aysan, 2017). Nevertheless, the stronger association revealed between the “high” SMA risk profile and shopping addictions (not always necessitating the internet), compared to other technology related addictions, challenges this conceptual distinction (Savci & Aysan, 2017). This finding may point to an expanding intersection between shopping and SMA, as an increasing number of social media platforms host easily accessible product and services advertising channels (e.g., Facebook property and car selling/marketing groups, Instagram shopping; Rose & Dhandayudham, 2014). In turn, the desire to shop may prompt a desire to find these services online, share shopping endeavors with others or find deals one can only access through social media creating a reciprocal effect (Rose & Dhandayudham, 2014). This possibility aligns with previous studies assuming reciprocal addictive co-occurrences (Tullett-Prado et al., 2021). This relationship might also be exacerbated by shared causal factors underpinning addictions in general, such as one’s drive for immediate gratification and/or impulsive tendencies (Andreassen et al., 2016; Niedermoser et al., 2021). Although such interpretations remain to be tested, the strong SMA and shopping addiction link evidenced suggests that clinicians should closely examine the shopping behaviors of those suffering from SMA behaviours, and if comorbidity is detected – address both addictions concurrently (Grant et al., 2010, Miller et al., 2019). Conclusively, despite some studies suggesting the distinction between technological, and especially internet related (e.g., SMA, internet gaming), addictions and other behavioral addictions (Gomez et al., 2022, Zarate et al., 2022), the current study’s high risk SMA profile associations appear not to differentiate based on the technological/internet nature that other addictions may involve.

Secondly, results suggest a novel hierarchical list of the types of addictions related to the higher SMA risk profile. While previous research has established links between various addictive behaviors and SMA (i.e., gaming and SMA; Wang et al., 2015), these have never before - to the best of the authors’ knowledge – been examined simultaneously allowing their comparison/ranking. Therefore, our findings may allow for more accurate predictions about the addictive comorbidities of SMA, aiding in SMA’s assessment and treatment. For example, Internet, shopping, gambling, gaming and sex addictions were all shown to more significantly associate with the high risk SMA profile than exercise and substance related addictive behaviors (King et al., 2014; Gainsbury et al., 2016a; Gainsbury et al., 2016b; Rose and Dhandayudham, 2014, Kamaruddin et al., 2018, Leung, 2014). Thus, clinicians working with those with SMA may wish to screen for gaming and sex addictions. Regardless of the underlying causes, this hierarchy provides the likelihood of one addiction precipitating and perpetuating another in a cyclical manner, guiding assessment, prevention, and intervention priorities of concurrent addictions.

Lastly, these results indicate a lower relevance of the high risk SMA profile with exercise/substance addictive behaviors. Considering excessive exercise, our study reinforces literature indicating decreased physical activity among SMA and problematic internet users in general (Anderson et al., 2017, Duradoni et al., 2020). Naturally, those suffering from SMA behaviours spend large amounts of time sedentary in front of a screen, precluding excessive physical activities. Similarly, the lack of a significant relationship between tobacco abuse and SMA has also been identified priori, perhaps due to the cultural divide between social media and smoking in terms of their acceptance by wider society and of the difference in their users (Spilkova et al., 2017). Contrary to expectations, there were weak/negligible associations between the high SMA risk profile with substance and alcohol abuse behaviours. This finding contradicts current knowledge supporting their frequent comorbidity (Grant et al., 2010, Spilkova et al., 2017; Winpenny et al., 2014). This finding may potentially be explained by individual differences between these users, as while one can assume many traits are shared between those vulnerable to substances and SMA, these may be expressed differently. For example, despite narcissism being a common addiction risk factor, its predictive power is mediated by reward sensitivity in SMA, where in alcoholism and substances, no such relationship exists (Lyvers et al., 2019). Perhaps the constant dopamine rewards and the addictive reward schedule of social media targets this vulnerability in a way that alcoholism does not. Overall, one could assume that the associations between SMA and less “traditionally” (i.e., substance related; Gomez et al., 2022) viewed addictions deserves more attention. Thus, future research is recommended.

### 4.3 Limitations and future direction

The current findings need to be considered in the light of various limitations. Firstly, limitations related to the cross-sectional, age specific and self-report surveyed data are present. These methodological restrictions do not allow for conclusions regarding the longitudinal and/or causal associations between different addictions, nor for generalization of the findings to different age groups, such as adolescents. Furthermore, the self-report questionnaires employed may accommodate subjectivity biases (e.g., subjective and/or false memory recollections; Hoerger & Currell, 2012; Sun & Zhang, 2020 The latter risk is reinforced by the non-inclusion of social desirability subscales in the current study, posing obstacles in ensuring participant responses are accurate.

Additionally, there is a conceptual overlap between SMA and Internet Addiction (IA), which operates as an umbrella construct inclusive of all online addictions (i.e., irrespective of the aspect of the Internet being abused; Anderson et al., 2017, Savci and Aysan, 2017). Thus, caution is warranted considering the interpretation of the SMA profiles and IA association, as SMA may constitute a specific subtype included under the IA umbrella (Savci & Aysan, 2017). However, one should also consider that: (a) SMA, as a particular IA subtype is not identical to IA (Pontes, & Griffiths, 2014); and (b) recent findings show that IA and addictive behaviours related to specific internet applications, such as SMA, could correlate with different types of electroencephalogram [EEG] activity, suggesting their neurophysiological distinction (e.g. gaming disorder patients experience raised delta and theta activity and reduced beta activity, while Internet addiction patients experience raised gamma and reduced beta and delta activity; Burleigh et al., 2020). Overall, these advocate in favour of a careful consideration of the SMA profiles and IA associations.

Finally, the role of demographic differences, related to one’s gender and age, which have been shown to mediate the relationship between social media engagement and symptoms of other psychiatric disorders (Andreassen et al., 2016) have not been attended here.

Thus, regarding the present findings and their limitations, future studies should focus on a number of key avenues; (1) achieving a more granular understanding of SMA’s associations with comorbid addictions via case study or longitudinal research (e.g., cross lag designs), (2) further clarifying the nature of the experience of SMA symptoms, (3) investigating the link between shopping addiction and SMA, as well as potential interventions that target both of these addictions simultaneously and, (4) attending to gender and age differences related to the different SMA risk profiles, as well as how these may associate with other addictions.

## 5 Conclusion

The present study bears significant implications for the way that SMA behaviours are assessed among adults in the community and subsequently addressed in adult clinical populations. By profiling the ways in which SMA symptoms are experienced, three groups of adult social media users, differing regarding the reported intensity of their SMA symptoms were revealed. These included the ‘low’ (52.4 %), ‘moderate’ (33.6 %) and ‘high’ (14 %) SMA risk profiles. The high SMA risk profile membership was strongly related to increased rates of reported internet and shopping related addictive behaviours, moderately associated with gaming, gambling and sex related addictive behaviours and weakly associated with alcohol, exercise and drug related addictive behaviours, to the point that such associations were negligible at most. These results enable a better understanding of those experiencing higher SMA behaviours, and the introduction of a risk hierarchy of SMA-addiction comorbidities that needs to be taken into consideration when assessing and/or treating those suffering from SMA symptoms. Specifically, SMA and its potential addictive behaviour comorbidities may be addressed with psychoeducation and risk management techniques in the context of SMA relapse prevention and intervention plans, with a greater emphasis on shopping and general internet addictive behaviours. Regarding epidemiological implications, the inclusion of 14 % of the sample in the high SMA risk profile implies that while social media use can be a risky experience, it should not be over-pathologized. More importantly, and provided that the present findings are reinforced by other studies, SMA awareness campaigns might need to be introduced, while regulating policies should concurrently address the risk for multiple addictions among those suffering from SMA behaviours.

Note 1: Firstly, results were compared across all converged models. In brief, the AIC and BIC are measures of the prediction error which penalize goodness of fit by the number of parameters to prevent overfit, models with lower scores are deemed better fitting (Tein et al., 2013). Of the 16 possible models, the parameterization with the most consistently low AIC’s and BIC’s across models with 1–8 profiles were chosen, eliminating 8 of the possible models. Subsequently, the remaining models were more closely examined through TIDYLPA using the compare solutions command, with the. BLMR operating as a direct comparison between 2 models (i.e. the model tested and a similar model with one profile less) on their relative fit using likelihood ratios. A BLMR based output p value will be obtained for each comparison pair with lower p-values corresponding to the greater fit among the models tested (i.e. if BLMR p >.05, the model with the higher number of profiles needs to be rejected; Tein et al., 2013). Entropy is an estimate of the probability that any one individual is correctly allocated in their profile/profile. Entropy ranges from 0 to 1 with higher scores corresponding with a better model (Tein et al., 2013; Larose et al., 2016). Finally, the N_min represents the minimum proportion of sample participants in any one presentation profile and aids in determining the interpretability/parsimony of a model. If N_min is 0, then there is a profile or profilees in the model empty of members. Thus, the interpretability and parsimony of the model is reduced (CRAN, 2021). These differing fit indices were weighed up against eachother in order to identify the best fitting model (Akogul & Erisoglu, 2017). This best fitting model was subsequently applied to the datasheet, and then the individual profilees examined through the use of descriptive statistics in order to identify their characteristics.

Note 2: With regards to the assumptions of the LPA Model, as a non-parametric test, no assumptions were made regarding the distribution of data. With regards to the subsequent ANOVA analyses, 2 assumptions were made as to the nature of the distribution. Homogeneity of variances and Normality. Thus, the distribution of the data was assessed via Jamovi. Skewness and Kurtosis for all measures employed in the ANOVA analyses. Skewness ranged from 0.673 to 2.49 for all variables bar the OGD-Q which had a skewness of 3.45. Kurtosis ranged from 0.11 to 6 for variables bar the OGD-Q which had a kurtosis of 13.9. Thus, all measures excepting the OGD-Q sat within the respective acceptable ranges of + 3 to −3 and + 10 to −10 recommended by Brown and Moore (2012).

Funding

Dr Vasileios Stavropoulos received funding by:

The Victoria University, Early Career Researcher Fund ECR 2020, number 68761601.

The Australian Research Council, Discovery Early Career Researcher Award, 2021, number DE210101107.

*Ethical Standards – Animal Rights*

All procedures performed in the study involving human participants were in accordance with the ethical standards of the institutional and/or national research committee and with the 1964 Helsinki declaration and its later amendments or comparable ethical standards. This article does not contain any studies with animals performed by any of the authors. Thus, the present study was approved by the Human Ethics Research Committee of Victoria University (Australia).

Informed consent

Informed consent was obtained from all individual participants included in the study.

Confirmation statement

Authors confirm that this paper has not been either previously published or submitted simultaneously for publication elsewhere.

Publication

Authors confirm that this paper is not under consideration for publication elsewhere. However, the authors do disclose that the paper has been considered elsewhere, advanced to the pre-print stage and then withdrawn.

Copyright

Authors assign copyright or license the publication rights in the present article.

Availability of data and materials

Data is deposited as a supplementary file with the current document.

## CRediT authorship contribution statement

**Deon Tullett-Prado:** Conceptualization, Methodology, Software, Validation, Formal analysis, Investigation, Data curation. **Vasileios Stavropoulos:** Supervision, Resources, Funding acquisition, Project administration. **Rapson Gomez:** Supervision, Resources. **Jo Doley:** Supervision, Resources.

## Declaration of Competing Interest

The authors declare that they have no known competing financial interests or personal relationships that could have appeared to influence the work reported in this paper.

## Data Availability

The data is made available via a link document.
