# Supplementary material for: Social media use and abuse: Different profiles of users and their associations with addictive behaviours
Source: Addict Behav Rep. 2023 Jan 21;17:100479. doi: 10.1016/j.abrep.2023.100479 (PMC9898019; doi:10.1016/j.abrep.2023.100479)
Supplement: Supplementary Data 1 [file mmc1.docx]

**Social Media Use and Abuse: Different Profiles of Users and Their Associations with Addictive Behaviours**

**Data, Syntax and Results**

Data in Brief

<https://github.com/disasterpasta/Social-Media-Use-and-Abuse-Different-Profiles-of-Users-and-Their-Associations-with-Addictive-Behavi/blob/main/Data%20in%20Brief.sav>

Syntax and Results

<https://github.com/disasterpasta/Social-Media-Use-and-Abuse-Different-Profiles-of-Users-and-Their-Associations-with-Addictive-Behavi/blob/main/Syntax%20and%20Results.docx>
